# Modelling Alzheimer’s disease in a dish: dissecting amyloid-β metabolism in human neurons

**DOI:** 10.1042/NS20230020

**Published:** 2024-01-11

**Authors:** Elizabeth Hill, Thomas J. Cunningham

**Affiliations:** MRC Prion Unit at UCL, Institute of Prion Diseases, Courtauld Building, London W1W 7FF, U.K.

**Keywords:** Alzheimers disease, amyloid beta, induced pluripotent stem cells

## Abstract

This scientific commentary refers to ‘Inhibition of insulin-degrading enzyme in human neurons promotes amyloid-β deposition’ by Rowland et al. (https://doi.org/10.1042/NS20230016). Insulin-degrading enzyme (IDE) and neprilysin (NEP) have been proposed as two Aβ-degrading enzymes supported by human genetics and *in vivo* data. Rowland et al. provide complementary evidence of a key role for IDE in Aβ metabolism in human-induced pluripotent stem cell (iPSC)-derived cortical neurons.

## The value of a complementary mechanistic approach to manipulate Aβ deposition

Alzheimer’s disease (AD) is a devastating, progressive neurodegenerative disease characterised by the aggregation and deposition of Aβ to form Aβ plaques in the brain. This has been proposed as the initiation event triggering downstream biochemical and cellular dysfunction ultimately leading to the clinical phase of the disease. Although we are living in an exciting age with disease-modifying Aβ-targeting immunotherapies reaching the clinic, the effects are modest and are associated with severe side effects in some patients. Whilst we may expect improvements to these treatments over time, there nevertheless remains an unmet need to substantially reduce the progression of neurodegeneration in AD, with a view to developing complementary approaches promoting Aβ degradation.

## Previous evidence for IDE and NEP involvement in Aβ metabolism

Aβ clearance is mediated through proteolytic degradation, which is driven by the action of multiple proteases. Two such enzymes with a strong evidence base as being the key players involved are the zinc metalloproteases insulin-degrading enzyme (IDE) and neprilysin (NEP).

Human genetic studies have implicated IDE in late-onset AD [1] and age of AD onset [2]. Extracellular Aβ has also been shown to be modulated by IDE-mediated proteolysis in primary rat cortical neurons [3], whilst IDE has been shown to be a major Aβ-degrading enzyme *in vivo* with *Ide^−/−^* mice reported to have a higher load of endogenous Aβ, albeit modest [4]. Additionally, IDE overexpression in APP transgenic mice lowered brain Aβ levels abrogating Aβ plaque formation [5].

There is also a wide range of literature supporting the role of NEP in Aβ clearance. NEP levels and activity are reportedly lower in AD patient brain (reviewed in [6]) and there is human genetics evidence that variants at the *MME* locus (encoding NEP) increase AD risk in certain human populations [7,8]. Interestingly, AD risk is further increased if individuals simultaneously harbour risk variants at the *IDE* locus [9]. There is also substantial *in vivo* evidence for a role of NEP in Aβ metabolism with mice lacking NEP exhibiting an increase in Aβ [10]. Concurrently, overexpression of NEP in AD mouse models has been shown to lead to lower brain Aβ levels, reduced plaques and increased survival [5,11–13].

Taken together, these and similar studies provide strong evidence for a role of IDE and NEP in Aβ degradation. However, one question that remains unaddressed is whether these enzymes contribute to Aβ metabolism specifically in human neurons, which would provide complementary data to the evidence base.

## IDE modulates Aβ clearance in human iPSC-derived neurons

Rowland et al. [14] provide complementary evidence to extend this work with the authors demonstrating that IDE is the major contributor to Aβ degradation in human-induced pluripotent stem cell (iPSC)-derived cortical neurons. They not only show this in human iPSC-derived neuronal lysates, but further validated these findings using an elegant 3D extracellular matrix (ECM) model with embedded human iPSC-derived cortical neurons, allowing the visualisation of Aβ deposition with the resulting plaques showing similar immunological properties to deposits in human AD brain. This experimental iPSC model was derived from individuals without AD and without familial AD mutations and interestingly shows a low baseline level of plaque formation.

They provide convincing evidence that IDE inhibition induced by three different inhibitors with distinct mechanisms of action promoted Aβ deposition in neurons derived from two independent iPSC lines. Considering the IDE inhibitors reduced Aβ degradation by >65%, this clearly nominates IDE as the predominant Aβ-degrading protease in this system, providing experimental support that enhancing IDE activity could be harnessed therapeutically as a complementary Aβ-lowering approach in human neurons.

## No effect of NEP inhibition on Aβ metabolism in human iPSC-derived neurons

Despite the strong evidence for a role of NEP in Aβ metabolism from *in vivo* studies, Rowland et al. [14] show that NEP does not appear to have a major role in Aβ degradation in iPSC-derived cortical neurons. Notably, NEP has been reported to robustly degrade disease-associated oligomeric forms of Aβ40 as well as oligomeric forms of Aβ42 *in vitro* [15]. Therefore, further work is warranted to investigate metabolism of longer, more aggregation prone Aβ forms in the human cellular context, including using models more relevant to the disease context such as using iPSCs derived from aged AD patients or patients with familial AD mutations. Finally, it is well established that non-neuronal cell types provide significant contributions in neurodegeneration; thus, further work is, therefore, also needed to decipher Aβ metabolism in more diverse human cellular contexts.

## Implications for therapeutic strategies

This study provides a human neuronal cellular context promoting the case for modulating IDE activity as a therapeutic strategy for AD and patients with cerebral β-amyloid angiopathy. Promoting Aβ clearance via modulating endogenous metabolic pathways has the potential to clear build-up of abnormal pathological Aβ assemblies and/or to slow the progress of pathological Aβ templated misfolding and propagation via lowering levels of non-disease associated Aβ substrate. The capacity to visualise and quantify Aβ in this system provides a platform for investigating a wider scope of disease contexts and modulating factors for Aβ lowering.

## Data Availability

NA

## Open Access

Open access for this article was enabled by the participation of University College London in an all-inclusive *Read & Publish* agreement with Portland Press and the Biochemical Society under a transformative agreement with JISC.

## Abbreviations

AD: Alzheimer’s disease
ECM: extracellular matrix
IDE: insulin-degrading enzyme
iPSC: induced pluripotent stem cell
NEP: neprilysin
